# Active Wavelength Control of Fiber Bragg Gratings: A Systematic Review of Tuning Mechanisms, Emerging Applications, and Future Frontiers

**DOI:** 10.3390/mi17020263

**Published:** 2026-02-19

**Authors:** Xiaoyan Wang, Erdong Xia, Chunrong Wang, Wen Ren

**Affiliations:** 1 School of Mechanical and Electrical Engineering, Sanming University, Sanming 365004, China; 20120933@fjsmu.edu.cn (E.X.); 20120928@fjsmu.edu.cn (C.W.); rw@fjsmu.edu.cn (W.R.); 2 Key Laboratory of Equipment Intelligent Control of Fujian Higher Education Institute, Sanming University, Sanming 365004, China

**Keywords:** fiber Bragg gratings, FBGs, active wavelength control, tuning mechanisms, tunable lasers, microwave photonics, quantum photonics, biomedical imaging, artificial intelligence

## Abstract

Fiber Bragg gratings (FBGs) have evolved from passive sensing elements into actively programmable photonic components, enabling dynamic wavelength control across diverse applications. This review provides a comprehensive and systematic overview of active wavelength control technologies for FBGs, deliberately excluding passive sensing applications. We systematically categorize the fundamental tuning mechanisms—including mechanical, thermal, optothermal, electro-optic, nonlinear optical, and hybrid approaches—and compare their performance characteristics in terms of tuning range, speed, precision, and trade-offs. Key enhancement techniques, such as mechanical amplification, thermal packaging, femtosecond laser fabrication, and FPGA-based interrogation, are examined. The transformative impact of actively controlled FBGs is elucidated across three major application domains: tunable and narrow-linewidth fiber lasers, reconfigurable microwave photonic systems, and emerging fields including quantum information processing and biomedical imaging. A consolidated technology map visualizes the connections between enabling techniques and applications. Finally, we critically analyze core challenges—performance trade-offs, control complexity, and integration bottlenecks—and outline future research directions driven by novel materials, artificial intelligence, and quantum technologies. This review offers a structured framework for understanding active FBGs as programmable photonic primitives, providing actionable insights for researchers and engineers in academia and industry.

## 1. Introduction

Fiber Bragg gratings (FBGs) stand as one of the most representative devices in fiber photonics, with their core functionality originating from the mode coupling effect induced by the periodic refractive index modulation within the optical fiber core. Since the first demonstration of photosensitivity in optical fibers and the fabrication of the first fiber Bragg grating by Hill et al. in 1978 [1], FBG technology has evolved into a cornerstone of modern photonics, enabling transformative applications in telecommunications, sensing, and laser systems. This physical mechanism endows FBGs with two fundamental characteristics: first, the passive response of their Bragg wavelength ($\lambda_{B}=2n_{eff}\Lambda$) to external environments (e.g., temperature, strain), making them excellent sensing elements; second, the dynamic controllability of the Bragg wavelength through the active manipulation of the grating period Λ or the effective refractive index $n_{eff}$ by external fields. While decades of research have thoroughly explored and utilized the former, establishing a mature technological system for FBG sensing, a parallel and increasingly important research direction—employing FBGs as actively programmable photonic core components for constructing dynamically reconfigurable optical systems—is demonstrating significant application potential and academic value. This is the specific paradigm upon which this review focuses.

In the current landscape where optical systems are evolving towards intelligence, reconfigurability, and high precision, the ability to dynamically and precisely control the optical wavelength/frequency domain has become paramount. Actively wavelength-controlled FBGs provide a critical technological pathway for this purpose. Through physical fields such as mechanical, thermal, electrical, or optical stimuli, the reflection or transmission spectrum of an FBG can be tuned, shaped, and programmed in real-time, enabling diverse functions like tunable filtering, dynamic dispersion management, wavelength routing, and laser frequency stabilization within a single device. This “software-defined” photonic functionality transcends the limitations of traditional static optical elements, offering an enabling core technology for next-generation optical communications, laser technologies, quantum information processing, and advanced sensing systems. Despite significant progress in active control technologies based on FBGs and their role in fostering innovative applications across several frontiers, the field still lacks a systematic consolidation framework. Existing literature often reports on specific tuning mechanisms or single application scenarios, lacking comparative analysis of different technological paths, knowledge connections across application domains, and a global perspective on the fundamental challenges and future development trends.

Therefore, this review aims to fill this gap by providing the first comprehensive and in-depth dedicated overview of active wavelength control technology for fiber Bragg gratings. This article is strictly confined to the active control paradigm; all research related to passive sensing is excluded from discussion. The main content of this review is structured as follows: First, we will systematically categorize and compare the fundamental physical mechanisms of active tuning (e.g., axial strain, thermal effect, electro-optic effect, optothermal effect, etc.) and their performance limits, while detailing key enhancement techniques developed to improve tuning range, speed, accuracy, and linearity. Subsequently, the article will focus on elucidating the groundbreaking applications of actively controlled FBGs in several cutting-edge fields, including but not limited to tunable/narrow-linewidth fiber lasers, reconfigurable microwave photonic filtering and signal processing, and their unique value emerging in interdisciplinary fields such as quantum information systems and advanced biomedical imaging. Building on a systematic review of the current technological state-of-the-art, the article will critically analyze the core challenges facing the field, such as performance trade-offs, control complexity, and integration bottlenecks. Finally, the article will propose forward-looking future research directions, including novel material and hybrid tuning platforms, artificial intelligence-driven intelligent tuning, fully programmable photonic systems, and Bragg structures for quantum applications.

Through this structured discussion, this review aims not only to provide a clear technical overview and application guide for researchers in academia and industry but also to outline a future development roadmap for the field. It seeks to promote the evolution of FBGs from fixed-function optical components into intelligent, system-level programmable photonic core elements, thereby laying a solid foundation for the development of future optical systems.

## 2. Fundamental Tuning Mechanisms and Key Technological Pathways

### 2.1. Fundamental Tuning

**Figure 1:**
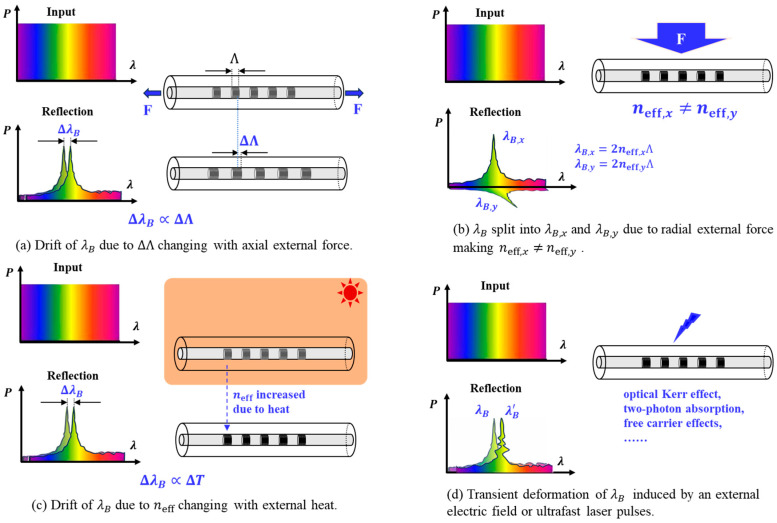
Classification and Principal Schematics of Active FBG Tuning Mechanisms.

The precise control of the Bragg wavelength ($\lambda_{B}=2n_{eff}\Lambda$) in active FBGs is achieved by externally altering the grating period (Λ) or the effective refractive index ($n_{eff}$) of the optical fiber. The pursuit of higher performance, such as broader tuning ranges, faster speeds, and narrower linewidths, driven by advancing applications in communications, sensing, and laser systems, has catalyzed the evolution of diverse tuning strategies. These strategies can be systematically categorized based on the nature of the external physical field applied, with each mechanism offering a distinct set of performance characteristics and trade-offs, as classified in Figure 1 and summarized in Table 1.

(1) Mechanical Tuning Mechanisms

Mechanical tuning methods induce wavelength shifts in FBGs by applying external forces to alter the physical grating period Λ (Figure 1a). Among these, axial strain tuning operates by subjecting the fiber to tension or compression, which directly modifies Λ through elastic deformation. Although the elastooptic effect concurrently induces a minor change in the effective refractive index $n_{eff}$, the resultant Bragg wavelength shift is predominantly governed by the change in Λ [2,3]. The relative shift follows the linear relation $\frac{\Delta \lambda_{B}}{\lambda_{B}}=\left(1-p_{e}\right)\varepsilon$, where ε is the axial strain and $p_{e}$ is the effective strain-optic coefficient, approximately 0.22 for germanosilicate fibers [4,5]. This approach provides a simple operating principle, a wide tuning range exceeding 40 nm, excellent linearity in the intrinsic strain-wavelength relationship, and millisecond-scale response. However, practical implementation with piezoelectric transducers introduces two distinct challenges: (i) actuator nonlinearities—hysteresis and creep that degrade precision and require closed-loop control for sub-picometer stability [6,7,8]; and (ii) reliability concerns—mechanical fatigue and risk of permanent damage under cyclic loading or over-strain conditions [6]. These limitations must be carefully managed in system design, particularly for long-lifetime or high-reliability applications.

An alternative configuration, bending-induced tuning, involves mounting or embedding the FBG within a flexible substrate such as a cantilever beam. Bending the structure transfers controlled axial strain to the grating, thereby modulating Λ. This method offers a straightforward and cost-effective means of achieving considerable wavelength shifts. Nevertheless, the setup can be relatively bulky, and the bending geometry may induce coupling to higher-order fiber modes, potentially leading to spectral distortion or broadening [9,10].

In transverse load tuning, force is applied perpendicular to the fiber axis. Here, the change in Λ via the Poisson effect is generally small. The dominant effect arises from the stress-induced birefringence, which splits the effective refractive index into two orthogonal polarization states ($n_{eff\_TE}$ and $n_{eff\_TM}$) and consequently divides the original Bragg peak into two distinct resonances (Figure 1b). This mechanism is particularly advantageous for vector force sensing and the realization of simple sideband filters. However, the inherent spectral splitting limits its utility for pure wavelength tuning, and the achievable tuning range remains modest while requiring careful control of the applied load [11,12].

(2) Thermal Tuning Mechanism

Thermal tuning achieves wavelength modulation in FBGs by exploiting temperature-induced material changes. The operating principle relies on two concomitant effects: the thermal expansion of the fiber, which alters the physical grating period Λ, and the thermo-optic effect, which modifies the effective refractive index $n_{eff}$. In silica fibers, the latter effect is dominant, contributing approximately 96% of the total wavelength shift (Figure 1c). The induced relative wavelength shift is quantitatively described by the relation $\frac{\Delta \lambda_{B}}{\lambda_{B}}=\left(\alpha +\xi \right)\Delta T$, where α is the thermal expansion coefficient and ξ is the thermo-optic coefficient. This yields a typical temperature sensitivity in the range of 10–20 pm/°C for wavelengths around 1550 nm [5].

Over a limited temperature range (e.g., −50 °C to 100 °C), the response can be approximated as linear with high accuracy, which underpins its reputation for excellent linearity in most practical applications. However, it is important to note that over extreme temperature spans, the thermo-optic coefficient ξ itself exhibits a weak temperature dependence, introducing an inherent nonlinearity [13,14,15]. This nonlinearity, while small, may require compensation in applications demanding ultra-high precision across broad thermal ranges, such as precision metrology or cryogenic quantum systems.

Beyond linearity considerations, the method offers high stability and the absence of moving parts, enabling very precise and smooth wavelength control. However, its practical application is constrained by a slow response time (on the order of seconds to minutes), relatively high power consumption, and a fundamentally restricted tuning range, which is ultimately bounded by the maximum safe operating temperature of the fiber materials and typically extends only to a few nanometers [13].

(3) Electromagnetic Tuning Mechanisms

Electromagnetic tuning methods modulate the Bragg wavelength of FBGs by applying external electric or magnetic fields to alter the effective refractive index $n_{eff}$. Electro-optic tuning, for instance, exploits the Pockels or Kerr effect, wherein an applied electric field directly modifies the material’s refractive index (Figure 1d). Since standard silica fiber exhibits a negligible linear electro-optic response, this approach necessitates the use of specialized fibers, such as polymer optical fibers, or hybrid integration with high-performance electro-optic crystals like lithium niobate (LiNbO_3_) [16]. While it offers the potential for exceptionally fast tuning speeds, extending into the gigahertz range, and is well-suited for high-speed optical modulation, its implementation is hindered by complex fabrication processes, high cost, low tuning efficiency, and poor compatibility with standard telecommunication fiber infrastructure.

In contrast, magneto-optic tuning operates via the Faraday effect, where an external magnetic field induces circular birefringence within the fiber. This creates a differential change in $n_{eff}$ for left- and right-hand circularly polarized light, typically manifesting as a splitting and shift of the original Bragg resonance [17,18]. The primary advantages of this method are its non-contact nature and direct applicability to magnetic field sensing. However, the effect is inherently weak in standard silica fibers, resulting in a very limited tuning range, unavoidable spectral peak splitting, and consequently, restricted practical utility for dedicated wavelength tuning applications.

(4) Optical Tuning Mechanisms

Optical tuning mechanisms represent a distinct class of methods for FBG wavelength control, wherein a pump light beam is employed to non-invasively modify the effective refractive index $n_{eff}$. The most prevalent technique is optothermal tuning (Figure 1c), which leverages the absorption of pump light, either by the FBG itself or a functional coating material such as graphene or a metal film. The subsequent localized heating induces a change in $n_{eff}$ predominantly via the thermo-optic effect [19]. This approach provides the significant benefits of non-contact, remote operation, inherent immunity to electromagnetic interference, good linearity, and high spectral resolution, which can reach on the order of 100 MHz. However, its performance is fundamentally limited by the dynamics of heat diffusion, resulting in response times on the millisecond scale and the potential for thermal hysteresis.

An alternative pathway is offered by photorefractive or nonlinear optical tuning (Figure 1d). In this method, an intense pump beam induces a direct, and often transient, alteration of the refractive index through nonlinear optical effects, such as the photorefractive effect, in specially engineered fibers or integrated waveguide materials [20]. This principle holds the promise of achieving extremely fast wavelength modulation, potentially on the nanosecond scale. The practical realization of this technique, however, faces substantial challenges, including the requirement for high optical pump power, increased system complexity, and the current relative immaturity of the technology compared to more established tuning methods.

(5) Hybrid Tuning Techniques

Hybrid tuning techniques are advanced methodologies that synergistically combine two or more fundamental physical mechanisms, such as mechanical, thermal, or optical effects, to achieve performance enhancements beyond the capabilities of any single method. A representative example is thermo-mechanical tuning, which typically involves mounting an FBG onto a composite structure, such as a piezoelectric tube. The application of voltage simultaneously induces a direct strain on the fiber via the piezoelectric effect and, through resistive heating, elevates its temperature. This combination leverages the large tuning range of the mechanical mechanism and the fine precision of thermal adjustment, enabling a synergistic and highly controlled wavelength shift [6]. The principal drawback of this integrated approach is the increased system complexity and the demand for more sophisticated multi-parameter control algorithms.

Another significant hybrid approach involves packaging the FBG with functional materials. In this configuration, the grating is embedded within or coated with materials that possess tailored properties, such as a high coefficient of thermal expansion or specific elastic moduli (e.g., certain metals or polymers). This engineered packaging acts as a transducer, which can amplify the FBG’s response to an external stimulus like pressure or temperature [21]. While this method offers the advantage of significantly enhanced sensitivity or the achievement of specialized functions like temperature compensation, it can also introduce nonlinearities into the system response. Furthermore, the packaging process itself may affect the long-term reliability and mechanical stability.

**Figure 2:**
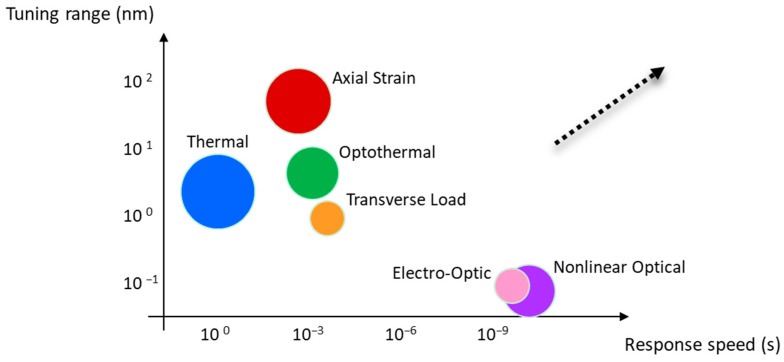
Performance landscape of active FBG tuning mechanisms: tuning range vs. response speed (bubble size indicates precision/linearity). The dashed arrow in the upper-right quadrant indicates the ideal design goal: achieving both wide tuning range and fast response simultaneously.

To provide a holistic and comparative view of the fundamental tuning mechanisms discussed above, Figure 2 presents a two-dimensional performance landscape plotting tuning range against response speed on logarithmic scales. This visualization reveals the distinct performance niches occupied by each mechanism and the inherent trade-offs between them.

Axial strain tuning (upper-left, large bubble) offers the widest tuning range (>40 nm) with good linearity, but its speed is limited to the millisecond regime by mechanical actuators such as piezoelectric transducers. In contrast, electro-optic and nonlinear optical tuning mechanisms (lower-right, medium bubbles) operate at the fastest speeds (ns to ps regime) but achieve only picometer-scale wavelength shifts, rendering them suitable for ultrafast modulation rather than broad-range tuning. Thermal tuning (center-left, large bubble) provides the highest precision and linearity with no moving parts, yet suffers from the slowest response (seconds) and moderate range (3–5 nm). Optothermal tuning (upper-center, medium bubble) offers an attractive compromise, achieving ranges up to 10 nm with millisecond response by leveraging localized photothermal heating. Transverse load (lower-center, small bubble) produces only sub-nanometer shifts and exhibits poor linearity due to birefringence-induced spectral splitting, making it more suitable for sensing than pure wavelength tuning. Bubble size in Figure 2 encodes relative precision/linearity, qualitatively derived from Table 1. Mechanisms with large bubbles (thermal, axial strain) are preferred for applications demanding high spectral fidelity, while small-bubble mechanisms (transverse load) are better suited for applications where range and speed are prioritized over precision. This performance landscape serves as a visual decision tool for selecting the appropriate tuning mechanism based on application-specific requirements. For example, swept-source OCT demands a wide range and moderate speed (favoring axial strain), while quantum frequency conversion requires ultra-high precision and stability (favoring thermal tuning). 

### 2.2. Key Technologies and Performance Enhancement

To meet the demands for dynamic, programmable, and high-precision wavelength control in applications such as tunable lasers and agile optical networks, researchers have developed a series of key techniques and performance enhancement strategies for active FBGs. These advancements systematically improve the tuning range, accuracy, response speed, and linearity of wavelength manipulation, forming the technical foundation for sophisticated active control systems. This section provides a detailed overview of these enabling technologies, as categorized in Table 2.

(1) Mechanical Tuning Enhancement Techniques

Mechanical tuning enhancement primarily focuses on structural optimization and novel actuation methods to improve efficiency, range, and speed. Elastic beam structure optimization involves the meticulous design of the shape, dimensions, and material of structures such as cantilever beams and simply supported beams. This can significantly improve the strain application efficiency and linearity for the FBG. For instance, an isosceles triangular cantilever can achieve a wider-range, more linear wavelength tuning while reducing unwanted chirp. Furthermore, a composite beam differential structure combining cantilever and simply supported beams enables the wavelengths of two FBGs to shift in opposite directions. This design not only enhances the efficiency of strain-based tuning but also inherently compensates for thermal drift, thereby improving the stability and repeatability of wavelength control under varying ambient temperatures [10]. To achieve a large wavelength tuning range with lower driving voltages, displacement amplification mechanisms based on the lever principle are employed. For example, a strain amplification structure, combined with a piezoelectric transducer driven by an optimized triangular waveform, can effectively extend the wavelength scanning range—reaching up to 6.3 nm—while also improving linearity and reducing nonlinear effects [22,23].

Beyond quasi-static tuning, high-frequency ultrasonic driving represents another strategy. Applying high-frequency ultrasonic waves (100 kHz–1 MHz) generated by a piezoelectric ceramic to an FBG can induce rapid fine-tuning of the grating period. This method not only dynamically alters the Bragg wavelength but also generates controllable sidebands in the reflection spectrum, making it suitable for high-speed optical modulation and signal processing [24,25].

(2) Thermal Tuning Enhancement Techniques

Thermal tuning strategies are enhanced through advanced packaging and localized heating methods to increase sensitivity and enable complex spectral shaping. One effective approach involves packaging the FBG within special materials possessing a high coefficient of thermal expansion, such as certain polymers or metals. This packaging allows temperature changes to induce greater effective thermal stress, thereby significantly enhancing the wavelength tuning sensitivity per unit temperature change. Research utilizing this technique has successfully increased the thermal tuning rate of external cavity lasers [26].

Another advanced method leverages the optothermal effect and local gradient field control. By utilizing the excellent photothermal conversion efficiency of novel nanomaterials (e.g., MXene Ti_3_C_2_T_x_), coated onto the FBG surface, a controllable gradient temperature field can be generated within the grating region through controlled illumination from a pump laser. This approach can dynamically induce and adjust the chirp of the fiber grating, enabling continuous, linear tuning of the reflection spectrum bandwidth and offering a novel pathway for all-optical control [27].

(3) Interrogation and Control Techniques

High-performance interrogation and control systems are essential for precise wavelength monitoring and feedback. High-precision interrogation techniques often rely on tunable Fabry–Perot filters as a core technology. By precisely controlling the cavity length of the Fabry–Perot filter via a piezoelectric ceramic for wavelength scanning, and combining this with advanced digital signal processing algorithms (e.g., local Gaussian fitting), high-resolution (up to the picometer level) wavelength demodulation over a wide dynamic range can be achieved [28].

For system integration and enhanced performance, advanced control and integration using a Field-Programmable Gate Array (FPGA) as the control core is highly effective. An FPGA-based system allows for the efficient integration of functions such as sawtooth wave signal generation, data acquisition, real-time processing, and communication. This highly integrated digital architecture effectively corrects nonlinear errors and compensates for temperature drift, substantially improving the overall speed, accuracy, and reliability of the interrogation system [29,30].

(4) Special Structures and Fabrication Techniques

Innovations in fabrication and novel structural designs create new possibilities for tuning mechanisms or providing a superior foundation for device performance.

Femtosecond laser direct writing technology is a groundbreaking FBG fabrication technique. It utilizes the nonlinear multiphoton absorption/ionization effect induced by ultrafast laser pulses to directly “inscribe” regions of refractive index modulation within the core of virtually any optical fiber, independent of the fiber’s intrinsic photosensitivity. This method offers exceptional thermal stability, as the induced refractive index change is a permanent structural modification that withstands higher annealing temperatures than UV-written gratings, extending the upper limit for thermal tuning [31,32,33]. It also provides true three-dimensional structural flexibility, enabling the fabrication of complex devices like tilted or phase-shifted gratings; tuning a phase-shifted grating, for instance, is applicable for high-precision filters or lasers. Furthermore, it allows seamless integration with specialty fibers, such as facilitating the writing of FBG arrays in multi-core fibers for space-division multiplexed applications [34,35].

Composite and microstructured packaging involves intentionally integrating the FBG into engineered materials or devices to impart or enhance specific tuning responses. This approach enables sensitivity amplification and functionalization; for example, packaging with a high-thermal-expansion material amplifies thermal sensitivity, while embedding within a shape memory alloy allows for large-range, discrete wavelength switching via phase transition [36]. Such packaging is also crucial for multi-parameter decoupling, ensuring pure tuning. An anisotropic micromechanical package can be designed to channel strain from an integrated actuator purely along the fiber axis while suppressing spurious bending or transverse stresses. This ensures predictable, linear Bragg wavelength shifts with minimal chirp, enhancing the purity and repeatability of the active tuning process [21].

Specialty fiber gratings themselves serve as novel tuning platforms. Fabricating FBGs on fibers with unique properties leads to distinct tuning characteristics determined by the fiber’s intrinsic attributes. Polymer optical fiber gratings leverage the significantly larger thermo-optic and elastooptic coefficients of polymer fibers, allowing the desired wavelength tuning range to be achieved with a much smaller stimulus, thereby improving energy efficiency and compactness [37,38]. Micro/nanofiber gratings exploit the strong evanescent field interaction in these fibers; their Bragg wavelength can be efficiently controlled by dynamically altering the refractive index of a functional coating or surrounding fluid, providing a pathway towards all-optical or microfluidic-driven tunable devices [39].

Through continuous innovation in mechanical structures, thermal management, interrogation algorithms, control systems, and fabrication techniques, the tuning performance of active FBGs has been comprehensively enhanced. The combination of these technological advancements forms a solid technical foundation for high-performance tunable FBG devices, paving the way for their promising applications in precision sensing, high-speed communications, and intelligent photonic networks.

## 3. Application Field: Tunable Fiber Lasers

The development of tunable fiber lasers demands precise and dynamic control over the emission wavelength, a requirement for applications ranging from spectroscopy to optical sensing. Actively FBGs have emerged as a foundational technology to meet this demand, providing a versatile and effective means for achieving wavelength agility. The following analysis synthesizes the fundamental principles and showcases their implementation through representative, high-impact case studies from recent literature.

### 3.1. Enabling Wavelength Agility and Tuning

FBGs serve as the cornerstone for wavelength agility in modern fiber lasers, enabling dynamic and precise emission control essential for spectroscopy, sensing, and communications. The core principle relies on actively perturbing the FBG’s Bragg condition $\left(\lambda_{B}=2n_{eff}\Lambda \right)$ to shift its reflection peak. Early implementations centered on external-cavity laser architectures, where an FBG acts as a tunable end-mirror for a broadband gain chip. Tuning was achieved mechanically via microelectromechanical systems (MEMSs) for wide ranges [40], or thermally via the thermo-optic effect for continuous adjustment. Advanced thermal designs, such as FBGs with engineered coatings, have achieved high tuning sensitivities of 65 pm/°C for compact, linear control [41].

The pursuit of all-fiber, integrated solutions led to the development of distributed Bragg reflector (DBR) and distributed feedback fiber lasers, where the grating is written into or adjacent to the gain medium. Uniform tuning via temperature or strain provides robust operation. A significant innovation for DBR lasers employs chirped and phase-sampled FBGs (CFBGs) in a Vernier configuration, enabling wide tuning ranges (e.g., 30 nm) with simplified control [42]. Beyond these established methods, novel materials and mechanisms are breaking traditional performance trade-offs. Integrating two-dimensional materials or phase-change materials with FBGs allows for ultrafast or non-volatile tuning. For example, coating a long-period grating with carbon nanotubes creates an all-optical tunable filter, enabling 16 nm of wavelength shift in a mode-locked laser via external light control [43]. Similarly, disordered FBG arrays fabricated by femtosecond lasers introduce random feedback, resulting in widely tunable (35.9 nm), single-mode random fiber lasers with sub-kHz linewidths [7].

For applications demanding both tunability and supreme spectral purity, advanced intracavity filtering techniques are critical. Integrating a chirped CFBG can enforce single-longitudinal-mode (SLM) operation over a broad band by creating multiple sub-cavities that suppress mode competition, demonstrated across the C-band with linewidths below 8.5 kHz [44]. Concurrently, the field is advancing towards intelligent control to overcome actuator nonlinearities. Machine learning-assisted inverse models and closed-loop feedback systems are being developed to achieve precise, hysteresis-compensated tuning and robust wavelength locking in the presence of environmental disturbances, moving the technology towards autonomous operation [45]. In summary, the evolution of FBG-based tunable lasers—from mechanical external cavities to intelligently controlled, all-fiber integrated systems—has yielded devices that combine wide tuning, narrow linewidth, and high stability, cementing their vital role in advanced photonics.

### 3.2. Achieving Kilohertz-Level Linewidth Stabilization

Beyond achieving wavelength tunability, attaining and maintaining an ultra-narrow, stable spectral linewidth is a paramount requirement for applications such as coherent optical communications, precision metrology, and quantum technologies. Fiber Bragg gratings are instrumental in this pursuit, primarily by serving as the critical spectral filter within the laser cavity to enforce single-longitudinal-mode (SLM) operation and suppress phase noise. The integration of FBGs into specialized cavity designs has enabled the realization of fiber lasers with linewidths consistently pushed into the sub-kilohertz regime.

**Figure 3:**
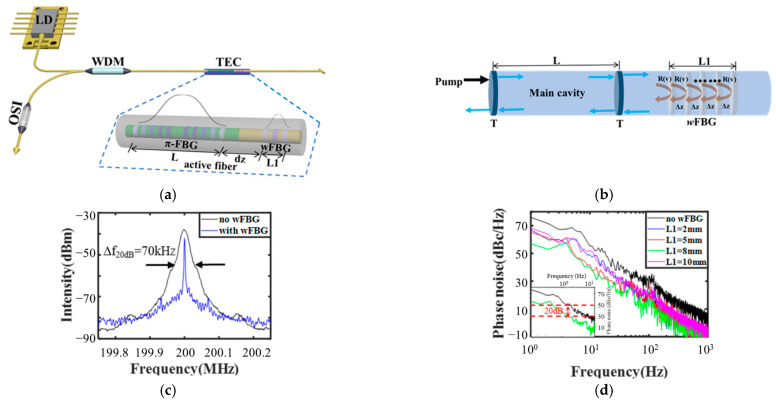
Self-injection locked narrow-linewidth fiber laser using a weak fiber Bragg grating. (**a**) Schematic diagram of the laser configuration. (**b**) Theoretical model of linewidth compression. (**c**) Measured laser linewidth showing compression from 70 kHz to 300 Hz with an 8 mm wFBG. (**d**) Phase noise power spectral density comparison, showing nearly 20 dB suppression in the 0–100 Hz range. Reprinted with permission from Ref. [46], © 2024 Elsevier.

A foundational and highly effective approach is the self-injection locking technique. Figure 3a illustrates the typical configuration of such a laser, where a π-phase-shifted FBG (π-FBG) forms the main cavity and a weak FBG (wFBG) written in the same fiber provides distributed feedback. As shown in the theoretical model in Figure 3b, the wFBG continuously reflects a portion of the output light back into the main cavity, effectively increasing the photon lifetime and suppressing spontaneous emission noise [46,47]. This process dramatically narrows the laser linewidth by suppressing relaxation oscillation noise and stabilizing the emission frequency. Experimental implementations using π-phase-shifted FBGs have demonstrated linewidth compression from the MHz range down to approximately 350 Hz, alongside significant reductions in relative intensity noise [46]. Extensions of this principle, utilizing simple weak FBGs, have further simplified the architecture while achieving linewidths as low as 300 Hz, as shown in Figure 3c [47]. For the most extreme spectral purity, ultra-short cavity DBR fiber lasers with cavity lengths on the order of millimeters leverage the inherently large longitudinal mode spacing to guarantee SLM operation. Such compact lasers, incorporating directly written FBGs in doped fiber, have achieved fundamental linewidths as low as 220 Hz [48].

To ensure robust SLM operation in longer, more conventional ring or linear cavities, advanced intracavity spectral filtering strategies are employed. Incorporating a CFBG capitalizes on its dispersive properties to create multiple sub-cavities with distinct effective lengths. This Vernier-like effect significantly increases the effective free spectral range, effectively suppressing multi-mode competition and enabling SLM operation across a wide tuning range with linewidths below 8.5 kHz [44]. An alternative and powerful method involves integrating a saturable absorber mechanism. A common implementation uses a section of unpumped gain fiber in conjunction with a narrowband FBG (e.g., a Fabry–Perot FBG). The dynamically induced grating within the saturable absorber provides ultra-narrowband loss modulation, effectively filtering out all but one longitudinal mode. This approach has proven highly effective, yielding stable SLM outputs with linewidths on the order of 550 Hz [49]. Furthermore, novel cavity designs like the Nested Fiber Ring function as high-quality passive mode filters, working in concert with saturable absorbers to guarantee exceptional SLM stability and optical signal-to-noise ratio.

The pursuit of narrow linewidth is also converging with the demand for wide tunability, as seen in systems like widely tunable random fiber lasers. By employing a disordered FBG array as the randomly distributed feedback element, these lasers can achieve a remarkable combination of a 35.9 nm tuning range and a minimum linewidth of 470 Hz [7]. This synergy demonstrates that spectral purity and agility are not mutually exclusive. Looking forward, the field is increasingly focused on intelligent stabilization. Machine learning algorithms are being explored to model and compensate for the complex, nonlinear dynamics of the laser cavity and its environment, promising enhanced long-term frequency stability and resilience against acoustic and thermal perturbations, thereby moving towards truly autonomous, ultra-stable laser sources.

### 3.3. Toward High-Power and Robust Single-Frequency Operation

Achieving high output power with robust single-frequency operation is crucial for applications like coherent beam combining and materials processing, yet it is fundamentally limited by nonlinear effects, primarily stimulated Brillouin scattering (SBS). Fiber Bragg gratings address this challenge by enabling high-fidelity single-frequency seed generation and facilitating nonlinear suppression in power amplification.

The foundational architecture for high-power, single-frequency sources is the master oscillator power amplifier. Here, the master oscillator is typically a low-noise, single-frequency fiber laser, often stabilized by the FBG-based narrow-linewidth techniques described in Section 3.2. The primary challenge lies in scaling the power of this pristine signal without triggering SBS or other detrimental effects like transverse mode instability. A straightforward and historically significant approach uses an FBG-based narrow-linewidth oscillator as the seed. This configuration benefits from the inherent simplicity and robustness of an all-fiber oscillator, achieving output powers of several hundred watts while maintaining narrow spectral bandwidths. For instance, a kW-level amplifier seeded by such an oscillator has demonstrated stable operation with a 0.08 nm linewidth [50]. Performance can be further optimized through cavity design; for example, an optimized composite FBG cavity with external feedback has been shown to improve temporal stability, enabling the subsequent amplifier stage to reach 6 kW with near-diffraction-limited beam quality [51].

To push the power ceiling further, active nonlinear suppression techniques are paramount. A key strategy involves spectral manipulation during amplification. While simple phase modulation broadens the linewidth to suppress SBS, it may compromise beam combining efficiency. Advanced co-modulation schemes, which combine precisely engineered amplitude and phase modulation, induce a controlled nonlinear spectral compression within the amplifier. This method actively narrows the spectrum as power increases, effectively raising the SBS threshold by approximately 1.7 times compared to traditional FBG-seeded schemes for the same output linewidth, enabling higher peak powers [52]. Other system-level optimizations, such as using specialty fibers with engineered acoustic profiles or strategically placing long passive fibers between the seed and amplifier to allow nonlinear spectral broadening, have proven effective in scaling continuous-wave and pulsed amplifiers to the multi-kilowatt regime [53,54].

Concurrently, there is a drive to enhance the power and robustness of the single-frequency oscillator itself. Integrating a saturable absorber within the oscillator cavity, often formed by an unpumped section of polarization-maintaining gain fiber in conjunction with a narrowband FBG, serves a dual purpose. It ensures stable SLM operation and can also contribute to nonlinear suppression within the oscillator stage, supporting higher intra-cavity powers. This approach has enabled the development of high-power, single-frequency pulsed sources, such as linearly polarized Q-switched fiber lasers, which are vital for applications like nonlinear frequency conversion [55]. Ultimately, the state-of-the-art demonstrates that through a synergistic combination of advanced FBG-based seed oscillators, intelligent nonlinear management in the amplifier chain, and innovative gain fiber designs, output powers exceeding 3 kW with narrow linewidths (~0.1 nm) and excellent beam quality are now achievable, marking a significant milestone in high-power photonics [51,53].

Design considerations for FBG-based tunable lasers: The three laser architectures discussed above address distinct application requirements. For applications prioritizing broad continuous tuning (e.g., swept-source OCT, spectroscopy), the Vernier-configuration DBR laser with chirped and phase-sampled FBGs [42] offers the widest range (up to 30 nm) but requires careful thermal stabilization. When ultra-narrow linewidth is paramount (e.g., coherent communications, precision metrology), the self-injection-locked DFB laser with weak FBG feedback [46,47] achieves sub-kHz linewidths at the cost of reduced tuning range. For high-power single-frequency operation (e.g., coherent beam combining, LIDAR), the MOPA architecture with FBG-based seed oscillator [50] provides kW-level output but demands sophisticated nonlinear suppression techniques. Emerging hybrid approaches combining multiple FBG structures with intelligent control [45] aim to simultaneously address these trade-offs, moving toward truly versatile laser sources.

## 4. Application Field: Microwave Photonics and Communication Networks

The unique capability of active FBGs to provide dynamic, precise, and wavelength-selective control has established them as enabling components in modern microwave photonic systems and advanced optical communication networks. Moving beyond static filtering, active FBGs introduce a layer of reconfigurability that is essential for adaptive signal processing, flexible resource allocation, and performance optimization in next-generation systems. This section delves into their critical roles in reconfigurable filtering, wavelength routing, and intelligent dispersion management.

### 4.1. Dynamic Spectral Filtering and Channel Management

In microwave photonics, which involves the generation, processing, and distribution of microwave signals using optical techniques, tunable filters are paramount. Active FBGs function as dynamic bandpass or band-stop filters, allowing for the selective processing of specific microwave channels carried on optical carriers.

The performance of these filters depends on the adopted technology and tuning mechanisms, with different mechanisms offering distinct advantages in various scenarios. Single-passband microwave photonic filters possess significant benefits such as a wide tuning range and high reconfigurability, making them highly attractive photonic subsystems in modern communication systems. A study [56] reported a novel tunable single-passband microwave photonic filter based on a MEMS platform, which employs a chirped fiber Bragg grating as the dispersive element instead of conventional single-mode optical fiber cables. Its tunability is achieved through a MEMS interferometer, with the center frequency tunable from 1.64 to 2.32 GHz and the 3-dB bandwidth ranging approximately from 280 to 300 MHz. Another work [57] proposed and validated a tunable single-passband microwave photonic filter with enhanced fineness. This high-Q single-passband filter is realized by cascading an optically injected semiconductor laser structure and an optoelectronic feedback resonator, enabling center frequency tuning from 10 to 18 GHz while maintaining a 3-dB bandwidth consistently below 4.97 MHz.

**Figure 4:**
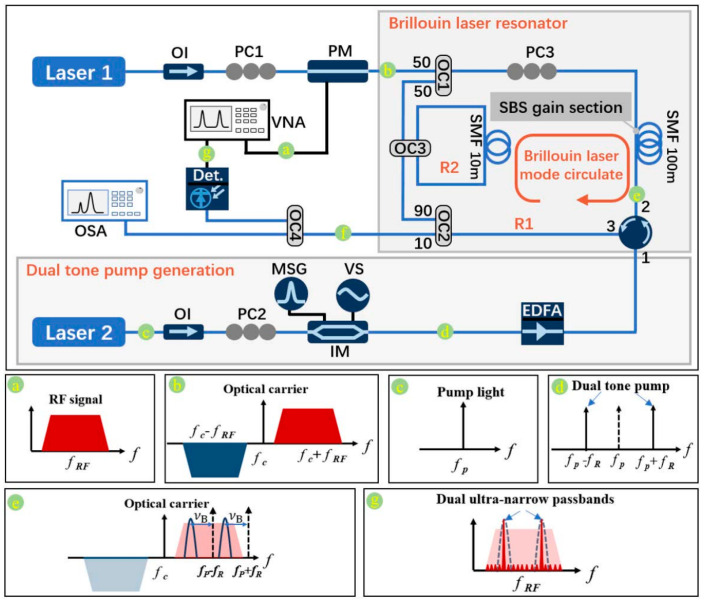
Experimental setup and operating principle of the dual ultra-narrow passband microwave photonic filter based on a dual-wavelength Brillouin laser [61]. (**a**) Schematic diagram of the proposed MPF. OC: optical coupler; OI: optical isolator; MSG: microwave signal generator; VS: voltage source; IM: intensity modulator; Cir: circulator; PM: phase modulator; PC: polarization controller; PD: photodetector; EDFA: erbium-doped fiber amplifier; SMF: single-mode fiber; VNA: vector network analyzer; OSA: optical spectrum analyzer. (**b**–**g**) Spectral evolution at key points: (**b**) phase-modulated double sideband signal; (**c**) pump light; (**d**) dual-tone pump after carrier-suppressed modulation; (**e**) interaction between modulated signal and Brillouin gain; (**f**) Brillouin gain spectra; (**g**) ultra-narrow dual passbands after Brillouin laser resonance. Reprinted with permission from Ref. [61] © 2024 Optica Publishing Group.

Regarding dual-wavelength tunable microwave photonic filters, a study [58] experimentally demonstrated a continuously tunable microwave bandpass filter implemented using an optical phase modulator and chirped fiber Bragg gratings. The filter tuning is accomplished by changing the optical carrier wavelength, which causes reflection at different physical locations in the linearly chirped fiber Bragg gratings. The experiment successfully demonstrated a two-tap microwave bandpass filter with a free spectral range tunable from 1.14 to 4.55 GHz. Another approach [59] employed a linearly chirped sampled Bragg grating structure incorporating two equivalent phase shifts to achieve a microwave photonic filter with dual independently tunable passbands, combined with microheaters to realize a tunable frequency range spanning from 37.2 to 186.1 GHz. A further study [60] proposed a flexibly tunable microwave photonic filter with dual ultra-narrow passbands based on a dual-wavelength narrow-linewidth Brillouin laser, achieving dual ultra-narrow passbands of 83 Hz, a maximum side-mode suppression ratio exceeding 28 dB, a maximum tuning range of the center frequency up to 20 GHz, and flexible passband interval tuning from 2 to 18 GHz. Figure 4 illustrates the experimental setup and operating principle of this MPF.

As shown in Figure 4a, the optical path consists of upper and lower branches. In the upper branch, an optical carrier from laser 1 is phase-modulated by the RF signal from a vector network analyzer, generating a double sideband signal (Figure 4b). In the lower branch, laser 2 outputs pump light, which is modulated by an intensity modulator driven by a microwave source to generate a dual-tone pump with carrier suppression (Figure 4c,d). The dual-tone pump is injected into a dual-ring Brillouin laser resonator (comprising a 100 m main fiber ring and a 10 m secondary ring), exciting two Brillouin gains (Figure 4f). These ultra-narrow Brillouin laser gains selectively amplify the modulated sideband signals, and after photodetection, yield two ultra-narrow filtering passbands with sub-kHz bandwidth (Figure 4g) [61]. By tuning the pump wavelength or the microwave frequency $f_{R}$, the center frequencies and interval of the dual passbands can be independently and continuously tuned, achieving the flexible filtering performance described above.

### 4.2. All-Optical Signal Processing and Computing

The active wavelength control technology for FBGs, when combined with inverse design methods, enables their evolution from conventional tunable filters into powerful programmable all-optical signal processors. By precisely designing the refractive index modulation of FBGs through advanced algorithms, their spectral responses can be directly mapped to specific mathematical operations or signal processing functions, allowing high-speed, broadband processing directly in the optical domain [61]. The core of this transformation lies in the inverse design and optimization tailored to target transfer functions.

To achieve complex processing functionalities, researchers employ optimization algorithms such as the quasi-Newton method to inversely derive the corresponding FBG structures based on target frequency-domain responses. For instance, photonic Hilbert transformers designed using transmission-type phase-modulated FBGs can accurately approximate ideal responses over bandwidths exceeding 500 GHz [62]. Similarly, by engineering the phase function of the reflection spectrum, all-optical fractional-order temporal differentiators with terahertz-level bandwidths can be constructed [63]. These designs have been experimentally validated, as demonstrated by the successful implementation of first-order optical differentiation [64]. The all-optical realization of these fundamental operations (Hilbert transformation, differentiation) provides key technologies for ultra-high-speed signal processing.

Furthermore, the capabilities of programmable FBGs can be extended to more advanced applications such as arbitrary optical pulse shaping. By mapping a target temporal waveform to its corresponding spectral response and inversely designing the FBG, precise and flexible manipulation of optical pulse waveforms can be achieved, with processing speeds reaching the sub-picosecond level [65]. This marks the transition of FBGs from implementing “standard computational units” to building “customizable signal synthesis engines.”

Notably, this programmable processing capability is extending into emerging fields such as quantum information technology. A representative application is the use of FBG pairs—designed based on Slepian sequences with orthogonal impulse responses—to implement quantum state encoding and decoding in high-dimensional quantum key distribution [66]. This approach enables both high spectral efficiency and high key generation rates in long-distance quantum communication, demonstrating the potential of FBGs in quantum information processing.

In summary, programmable FBGs have emerged as a versatile platform for all-optical signal processing. Looking ahead, with advancements in integrated photonics fabrication processes, programmable integrated Bragg gratings are expected to enable more complex photonic signal processing systems on a single chip, thereby playing an increasingly central role in future optical communication, optical computing, and quantum information processing networks.

### 4.3. Performance Optimization for Transmission Systems

Beyond enabling advanced all-optical signal processing, the programmable nature of FBGs, achieved through active wavelength control, plays an equally pivotal role in enhancing the performance and resilience of optical transmission systems themselves. Modern high-capacity links are plagued by a cascade of physical layer impairments, including chromatic dispersion, polarization-mode dispersion, erbium-doped fiber amplifier gain non-uniformity, and fiber nonlinearities. Static compensation modules are insufficient for dynamically reconfigurable networks or links experiencing time-varying conditions. Programmable FBGs offer a versatile toolkit for the dynamic, adaptive, and precise management of these impairments, thereby expanding system reach, capacity, and operational flexibility.

A cornerstone application is dynamic dispersion compensation, a critical requirement for high-bit-rate transmission. The ability to precisely tailor and tune the dispersion profile of a chirped FBG allows for real-time correction of accumulated chromatic dispersion across variable link lengths or in dynamically switched paths. Early system demonstrations at 40 Gb/s using tunable FBGs with distributed thin-film heaters showcased their capability to significantly widen the operational launch power window compared to static dispersion-compensating fiber, a direct indicator of enhanced system robustness [67]. Advances in fabrication, such as employing linearly chirped phase masks, have led to devices with improved performance metrics, including broader tuning ranges (~200 ps/nm) and reduced group delay ripple, enabling high-fidelity transmission with minimal power penalty [68]. More recently, strain-tuning mechanisms have been exploited to achieve an exceptionally wide dispersion tuning range from 800 to 2657 ps/nm in nonlinear chirped FBGs, with simulation results confirming excellent bit-error-rate performance over standard fiber spans, highlighting the potential for future agile dispersion management engines [69]. For even more complex linear impairments, FBG-based solutions extend to higher-order dispersion and polarization-mode dispersion. Sampled nonlinearly chirped FBGs have been employed for tunable multi-channel compensation in 40-Gb/s WDM systems, effectively managing dispersion slopes across several channels [70]. Furthermore, integrated devices featuring twin chirped FBGs have demonstrated the independent and simultaneous tuning of chromatic dispersion and differential group delay (a first-order polarization-mode dispersion metric), providing a compact solution for combating combined linear distortions in ultra-high-speed systems [71].

Concurrent with linear impairment correction, managing the spectral gain profile of optical amplifiers is essential for maintaining channel power uniformity in WDM systems. FBGs, particularly when used in cascaded configurations, serve as effective gain-flattening filters. Research has shown their superiority over conventional methods, with studies on different FBG host materials like alumino-germanosilicate demonstrating the ability to achieve high gain (30 dB) with excellent flatness (1% relative gain difference) over broad bandwidths [72]. The evolution from static flattening to dynamic control is exemplified by gain-clamped erbium-doped fiber amplifier designs incorporating FBGs. One recent architecture for the L-band utilizes an FBG and a broadband mirror to form a linear cavity, with a variable optical attenuator for loss control. This design achieves a wide 42 nm gain-clamped bandwidth with high stability (±0.25 dB), effectively mitigating gain transients caused by channel add/drop, thus ensuring consistent transmission quality [73].

Perhaps the most sophisticated role of programmable FBGs in performance optimization lies in the synergistic management of nonlinear effects, moving beyond mere compensation to proactive signal and link design. Intriguingly, the choice of dispersion compensation technology itself influences the nonlinear penalty. Comparative analysis has revealed that systems using chirped FBGs for dispersion compensation exhibit significantly suppressed cross-phase modulation compared to those using dispersion-compensating fiber, due to fundamental differences in their dispersion maps [74]. This indicates that FBG-based compensation offers inherent advantages for nonlinearity mitigation. More directly, the pulse-shaping capabilities of FBGs are strategically employed to precondition optical signals for enhanced nonlinear tolerance. By synthesizing advanced waveforms like Nyquist or parabolic pulses, which possess favorable spectral and temporal properties, FBG-based shapers can reduce intra-channel and inter-channel nonlinear interactions at the source [65,75]. This approach represents a paradigm shift from post-transmission correction to pre-transmission optimization.

Finally, the wavelength-selective programmability of FBGs naturally extends to the reconfigurable network nodes that interconnect transmission spans. Tunable optical add-drop multiplexers based on FBGs provide the granularity needed for dynamic wavelength routing, which is fundamental to flexible grid and elastic optical networks. Architectures utilizing multi-port circulators and narrowband FBGs offer low-loss and cost-effective solutions for dynamically managing channel traffic [76]. Similarly, tunable filters built from concatenated chirped long-period gratings enable precise channel isolation and selection, facilitating efficient bandwidth utilization and network reconfiguration [77]. These components ensure that the performance benefits achieved within the physical layer are seamlessly supported at the network switching layer.

In conclusion, FBG-based active wavelength control has established itself as an indispensable technology for holistic transmission system optimization. It addresses a comprehensive suite of challenges—from linear and nonlinear physical layer impairments to spectral gain management and network-layer flexibility—through dynamic dispersion compensators, intelligent gain equalizers, advanced pulse shapers, and reconfigurable filtering/routing elements. As optical networks evolve towards greater agility and intelligence, the integration of programmable FBGs with real-time monitoring and control algorithms will be crucial for realizing self-optimizing and ultra-high-capacity communication infrastructure.

## 5. Application Field—Other Emerging Application Fields

The application of active FBG wavelength control extends beyond conventional lasers and telecommunications, finding pivotal roles in several cutting-edge fields that demand dynamic optical manipulation. These applications leverage the unique capability of active FBGs to provide real-time, precise, and programmable control over light, opening new paradigms in quantum technologies, biomedical imaging, and advanced optical signal processing.

### 5.1. Quantum Information Systems: Enabling Precision and Stability

**Figure 5:**
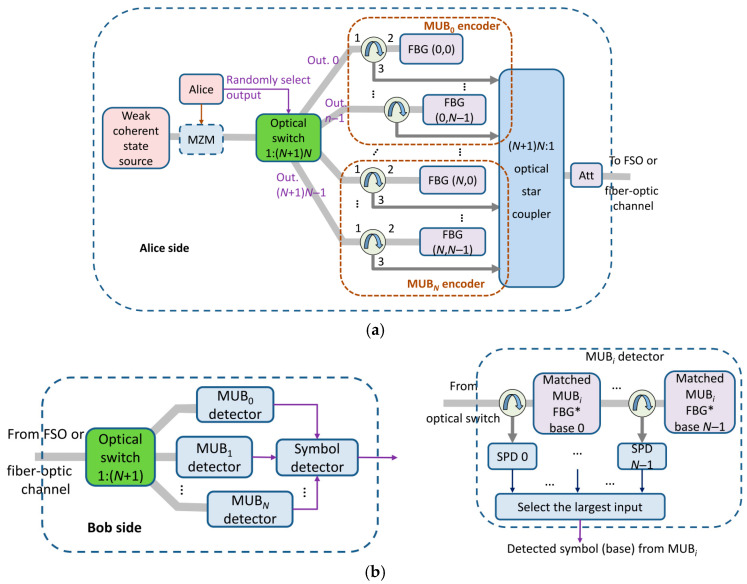
FBG-based encoder and decoder architectures for high-dimensional quantum key distribution. (**a**) Alice’s MUB-FBG encoder: an optical switch selects one of (N + 1)N FBGs, each with superimposed impulse responses representing a specific basis state within a MUB. (**b**) Bob’s MUB-FBG decoder: left part shows the overall decoding scheme, where a 1:(N + 1) optical switch randomly selects the measurement MUB; right part details the configuration of the i-th MUB detector, consisting of N complex-conjugate FBGs and SPDs. Only the FBG matching Alice’s encoding reflects the pulse, triggering the corresponding SPD and enabling symbol identification. Reprinted with permission from Ref. [67], © 2018 IEEE.

FBGs are evolving from mature components in classical optical communications into indispensable enabling devices for quantum information systems, such as quantum key distribution (QKD) and quantum networks. A paradigmatic example is the use of FBGs as programmable encoders and decoders for high-dimensional QKD, as illustrated in Figure 5.

As shown in Figure 5a, Alice’s encoder employs an optical switch to randomly select one of (N + 1)N FBGs, each fabricated with superimposed impulse responses derived from Slepian sequences. These FBGs represent specific basis states within mutually unbiased bases (MUBs), enabling the encoding of log_2_N bits per photon [67]. On the receiver side, Figure 5b presents Bob’s decoding architecture. The left part of Figure 5b shows the overall decoding scheme: a 1:(N + 1) optical switch randomly selects the measurement MUB. Once an MUB is selected, the corresponding detector bank, detailed in the right part of Figure 5b, performs the decoding. Here, a series of complex-conjugate FBGs are arranged in parallel; only the FBG whose impulse response is conjugate-matched to Alice’s encoding reflects the incoming pulse, triggering the corresponding single-photon detector (SPD) at circulator port 3. This architecture achieves high spectral efficiency and secret-key rates simultaneously, while imposing less stringent bandwidth requirements compared to time-frequency or time-bin protocols.

Beyond QKD, FBGs also serve as an integrated platform for efficient and stable quantum light sources. Quantum information processing imposes extremely stringent demands on photonic components, requiring exceptional wavelength selectivity, ultra-low insertion loss, superb phase stability, and inherent compatibility with fiber-optic networks. Traditional discrete optical elements face fundamental challenges in terms of complexity, stability, and scalability. In this context, FBGs offer a unique and powerful physical platform for constructing high-precision, highly stable, and scalable quantum hardware, leveraging their programmable spectral response, intrinsic low-loss all-fiber architecture, and compatibility with advanced fabrication processes.

As a Programmable Encoder/Decoder for High-Dimensional Quantum States, FBGs utilize their specific spectral responses to directly manipulate the quantum states of photons in the optical frequency domain. This signifies the evolution of FBGs from passive wavelength-selective elements to active programming tools capable of precisely synthesizing complex quantum operations, laying a hardware foundation for building high-dimensional, robust quantum communication systems.

As an Integrated Platform for Efficient and Stable Quantum Light Sources, the core function of FBGs—particularly their two-dimensional variant, circular Bragg gratings—is to serve as microcavities monolithically integrated with quantum emitters, such as semiconductor quantum dots, directly addressing the efficiency and stability challenges of quantum light sources. Through advanced inverse design and Bayesian optimization methods, the geometry of hybrid circular Bragg gratings can be globally optimized, simultaneously achieving a Purcell factor exceeding 20 and a direct fiber coupling efficiency greater than 86%, while accounting for fabrication tolerances [78]. Experimentally fabricated devices have demonstrated spontaneous emission lifetimes below 50 picoseconds and Purcell factors beyond 15, along with excellent temperature stability [79]. The value of this approach lies in its monolithic integration of three key functional units—single-photon generation (quantum dot), optical field control and enhancement (microcavity), and efficient fiber output—fundamentally avoiding the losses and instability associated with coupling discrete components. This provides a crucial solution for realizing “plug-and-play” deterministic quantum light sources. Concurrently, the trend towards modularization and all-fiber integration in quantum systems is evident, exemplified by fully fiber-coupled, alignment-free entangled photon-pair source modules whose design philosophy aligns closely with the goals of high stability and easy integration pursued by FBG-based sources [80].

As an Ultra-High-Precision Spectral Filter for Quantum Channels, FBGs address the core challenge of noise suppression in quantum signal transmission. At the quantum receiver or within quantum networks, it is imperative to filter out noise photons with extreme efficiency to enhance the signal-to-noise ratio and protocol security. Through sophisticated inverse design, introducing multiple π-phase discontinuities into an FBG enables the fabrication of filters with remarkable performance. Such devices can achieve a deep suppression of (128 ± 6) dB and an extremely steep roll-off of (90.6 ± 0.7) dB/GHz [81]. This performance far surpasses that of traditional filters, demonstrating that FBG-based devices can meet the stringent demands of quantum networks for ultra-narrowband, ultra-high suppression ratio, and low-loss filtering, making them essential components for constructing long-distance, high-fidelity quantum channels.

In summary, through their unique capabilities in spectral precision programming, optical field localization and extraction, and extreme filtering, fiber Bragg gratings are undergoing a transition from being “functional components” to becoming “system cornerstones” at multiple critical nodes within quantum information systems—from the preparation and manipulation of quantum states to their transmission and measurement. Looking forward, with the broader application of inverse design and intelligent optimization algorithms, and the continued drive towards multifunctional quantum photonic integrated circuits, FBG technology is poised to further advance quantum hardware towards higher performance, greater reliability, and enhanced modularity, accelerating the practical realization of quantum information technologies.

### 5.2. Advanced Biomedical Imaging and Sensing: Toward Programmability

While passive FBGs are staples in physical sensing, their active counterparts are enabling new functionalities in biomedical applications that require wavelength agility.

A typical example of this paradigm is evident in high-speed, broadband Optical Coherence Tomography, especially in spectral bands of high biomedical relevance. Swept-source Optical Coherence Tomography requires lasers capable of rapidly and linearly scanning a broad spectral bandwidth. Traditional methods using bulk optics or dispersive fibers are limited in terms of power efficiency, stability, and compactness, particularly in the 2-micron and 1060 nm spectral windows beneficial for imaging highly scattering tissues and the retina, respectively. Here, CFBGs emerge as an exceptional programmable solution. As highly dispersive, all-fiber “time-stretch” elements, CFBGs can temporally disperse the broadband spectrum of mode-locked femtosecond pulses. In the 2-micron band, compared to dispersive fibers, CFBGs offer outstanding power efficiency and temporal stability, enabling the construction of a robust all-fiber swept source with a 33 nm bandwidth, an 18.4 MHz sweep rate, and watt-level output power, suitable for deep-tissue Optical Coherence Tomography [82]. Similarly, for retinal imaging at 1060 nm, the use of a long CFBG in conjunction with an all-normal-dispersion supercontinuum source enables a 90 nm sweep at a 10 MHz rate with a 93% duty cycle and extremely low noise. This configuration achieves a sensitivity of 84 dB, approaching the shot-noise limit, directly translating into higher-quality in vivo retinal images [83]. In both cases, the programmable dispersion characteristics of CFBGs serve not only as a compensator but also as a key component for customizing the sweep characteristics (speed, linearity, bandwidth) of the light source to achieve optimal imaging performance in specific biological windows.

Meanwhile, in the field of dynamic physical sensing, the concept of programmability extends to high-speed demodulation and intelligent network design. Modern structural health monitoring demands the detection of high-frequency dynamic strains (such as those from impacts or vibrations) with high sensitivity across distributed sensor arrays. This requires a shift from static wavelength demodulation to active, high-speed addressing of FBG spectral signatures. Advanced demodulation systems now employ tunable lasers, interferometric schemes, and arrayed waveguide gratings to achieve MHz-rate sampling of FBG wavelength shifts, enabling precise capture of transient events [84]. Furthermore, the topology of sensor networks themselves is becoming programmable. Recent demonstrations show that FBG sensors can be efficiently multiplexed in flexible tree topologies rather than simple serial arrays. Using directly modulated laser arrays for heterodyne detection, sensors within multi-stage tree networks can be distinguished spectrally and temporally with high signal-to-noise ratios. This architecture supports long-distance demodulation (e.g., 20 km) and allows for the deployment of scalable, reconfigurable sensors to optimize large-scale infrastructure [85]. The integration of adaptive algorithms, such as time-frequency wavelet analysis for impact localization, further points toward intelligent, self-diagnosing sensing systems where the responses of the FBG network are dynamically processed and interpreted.

In conclusion, the progress of FBGs in the biomedical field is reflected in their ascent from simple sensing transducers to programmable core subsystems that define system-level capabilities. The convergence of these paths heralds a future where integrated programmable FBG platforms may form the foundation of multifunctional devices capable of simultaneous high-resolution imaging and multi-parameter dynamic sensing, all guided by adaptive control algorithms for personalized diagnostic and monitoring applications.

### 5.3. Consolidated Technology Landscape

**Figure 6:**
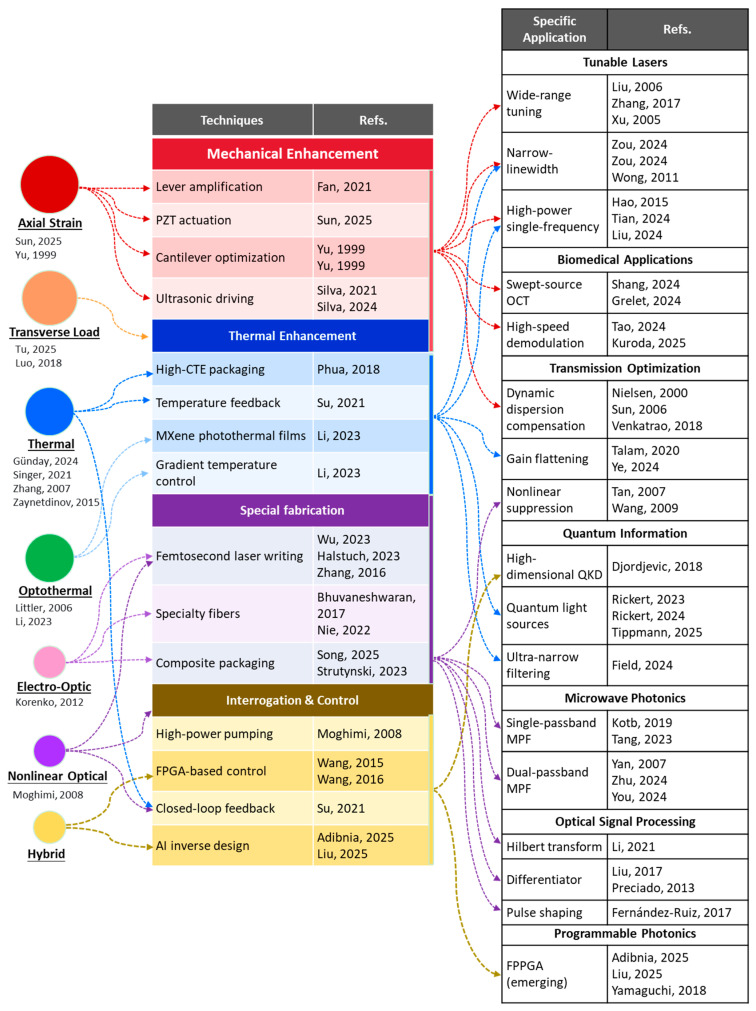
Mapping of enabling technologies to application domains for active FBG wavelength control. Bubble colors indicate technology maturity (larger bubbles represent more established technologies), and dashed arrows represent emerging or conceptual pathways. This map serves as a navigational tool to connect specific enabling technologies with their corresponding applications [5,6,10,11,12,13,14,15,16,19,20,21,22,25,26,27,28,30,31,32,33,34,37,38,40,41,42,43,46,47,49,50,52,55,56,57,58,59,61,62,63,64,65,66,67,68,71,72,73,74,77,78,79,80,81,82,83,84,86,87,88].

The applications discussed in Section 3, Section 4 and Section 5 are enabled by a diverse set of technologies. Figure 6 consolidates this landscape, mapping the flow from enabling technologies (left) to application domains (right).

As shown in Figure 6, enabling technologies (left column) are grouped into four categories—Mechanical Enhancement, Thermal Enhancement, Special Fabrication, and Interrogation & Control—with representative references in brackets. These technologies flow into seven thematic application groups on the right: tunable lasers (Section 3), microwave photonics and transmission systems (Section 4), and emerging fields including quantum information and biomedical imaging (Section 5). Bubble size qualitatively indicates technology maturity (larger bubbles represent more established techniques), and dashed arrows denote emerging or conceptual pathways. This map serves as a navigational tool to connect specific enabling technologies with their corresponding applications and reveals both the remarkable versatility of active FBGs and the open challenges that remain. Section 6 discusses future directions, building on this landscape.

## 6. Frontier Directions and Open Challenges

The future of active FBG technology lies in intelligentization, deep integration, and application-specific specialization. Key pathways—including novel materials, AI-driven control, programmable systems, and quantum-optimized components—promise to transform FBGs from fixed components into reconfigurable photonic building blocks, paving the way for “software-defined photonics.”

### 6.1. Core Challenges

The preceding analysis solidly positions active FBG wavelength-control technology as a cornerstone for next-generation photonics, enabling functionalities from tunable lasers and dynamic network management to quantum information processing [8,80,89]. This maturation, however, concurrently exposes fundamental limitations that arise from the material, control, and integration paradigms underpinning current implementations. The progression of applications towards higher speeds, broader operational bandwidths, and integration into complex systems magnifies these constraints, establishing a set of core challenges that must be addressed to unlock the full potential of programmable FBGs.

A primary and persistent challenge lies in the intrinsic performance trade-offs of established tuning mechanisms. Each prevalent technique—thermal, piezoelectric, and strain-based actuation—suffers from a compromise between tuning speed, achievable range, and power efficiency. Thermal tuning, while capable of substantial wavelength shifts, is fundamentally limited by the thermal mass of the system, resulting in slow (second-scale) responses and high static power consumption for maintaining a setpoint [6,30]. Piezoelectric actuation enables millisecond-scale tuning but is often constrained in range and plagued by nonlinear hysteresis and creep, degrading repeatability and long-term stability [41,90]. Although mechanical strain can offer a favorable combination of speed and range, it introduces concerns regarding packaging reliability, cyclical fatigue, and potential long-term drift, complicating its use in systems demanding unwavering precision.

These actuator-level imperfections give rise to a second-order challenge: the complexity of achieving precise, adaptive, and intelligent control. The nonlinear, hysteretic, and environmentally sensitive responses of common actuators render simple open-loop control schemes inadequate for applications requiring high spectral fidelity or dynamic tracking. Consequently, implementation often relies on application-specific, closed-loop feedback systems incorporating optical power monitors or wavelength meters. These solutions add complexity, cost, and bulk, and they typically lack the adaptability to autonomously optimize performance across varying operational conditions or compensate for aging effects [6,45]. The absence of a generalized, intelligent control framework is a significant bottleneck for the deployment of robust and “set-and-forget” programmable FBG subsystems.

A third systemic challenge is the bottleneck in integration and scalability. The actuation and packaging approaches for most tunable FBGs remain discrete and bulky, often involving external heaters, piezoelectric stacks, or mechanical transducers attached to the fiber. This paradigm is fundamentally at odds with the photonics industry’s drive towards miniaturization, low-cost wafer-scale manufacturing, and monolithic integration with silicon photonic or CMOS electronic platforms. The inability to co-fabricate high-performance, reliably actuated Bragg gratings alongside other active and passive photonic components on a common chip impedes the development of complex, scalable, and cost-effective programmable photonic integrated circuits [30,61,91,92].

Finally, emerging frontier applications are imposing new extreme-environment performance demands that stretch current technologies to their limits. Quantum information systems, for instance, may require ultra-stable FBG-based filters or cavities operating at cryogenic temperatures, where material properties and thermo-optic coefficients differ drastically from room temperature. Similarly, high-power, coherent LIDAR systems or precision metrology tools demand exceptional wavelength stability in the face of significant acoustic noise, vibration, or rapid thermal transients [26,33,93,94]. The performance of standard FBGs and their actuation mechanisms under such non-standard, harsh conditions is not yet fully characterized or optimized, representing a critical area for foundational investigation.

In summary, the advancement of active FBG technology is gated by a confluence of challenges spanning intrinsic actuator trade-offs, control intelligence, integration pathways, and performance in extreme environments. These interconnected issues define the present research frontier and provide the direct impetus for the innovative trends and directions discussed in the following section.

### 6.2. Future Trends and Research Directions

The future trajectory of active FBG technology is poised to shift from incremental refinement to transformative advancement, driven by convergence across multiple disciplines. To transcend the fundamental challenges outlined in Section 4.1, future research will focus on paradigm-shifting innovations in materials, control intelligence, and system architecture. These innovations promise to catalyze the development of a new generation of highly programmable photonic components. Supported by emerging high-impact research, the following sections outline the most promising pathways forward.

#### 6.2.1. New-Material and Hybrid Tuning Platforms

The quest for superior performance is catalyzing a transition from conventional actuation methods toward platforms that leverage novel materials and hybrid integration strategies. Advanced materials are at the forefront of this shift. For instance, two-dimensional materials like graphene exhibit exceptional electro-thermal properties, offering a pathway to ultrafast, nanosecond-scale thermal tuning with remarkably low power consumption [95,96]. Similarly, phase-change materials (PCMs) such as GSST could enable non-volatile, discrete wavelength switching with zero static power, representing a paradigm shift for designing ultra-low-power reconfigurable photonic circuits [94]. Concurrently, research into high-performance piezoelectric thin films (e.g., PMN-PT) aims to deliver more linear and efficient mechanical actuation with significantly reduced hysteresis compared to traditional bulk ceramics. Beyond single-mechanism enhancements, future platforms will explore synergistic multi-physics coupling. Engineered structures that combine, for example, the thermo-optic effect with the electro-optic effect could unlock complex, tailored tuning responses unattainable by any single mechanism [27,97]. Furthermore, the heterogeneous integration of FBG structures with silicon photonic platforms via advanced bonding techniques is a critical step toward realizing compact, low-cost, and highly functional photonic integrated circuits that harness the complementary strengths of diverse material systems [91,92].

#### 6.2.2. AI-Driven Intelligent Tuning

Artificial intelligence (AI) and machine learning (ML), in particular, are set to revolutionize the control and operational paradigm of active FBGs. This shift moves beyond mere precision tuning toward adaptive, intelligent systems capable of overcoming inherent nonlinear dynamics. A key application lies in leveraging deep learning for inverse modeling and dynamic compensation. Instead of relying on imperfect analytical models, deep neural networks (DNNs) can be trained to learn the complex, nonlinear mapping between control inputs (e.g., applied voltage) and the resulting spectral response of the FBG. This data-driven approach effectively creates a real-time inverse model that can compensate for hysteresis, creep, and environmental cross-sensitivity, enabling highly accurate open-loop control, as evidenced in preliminary studies on piezoelectric actuators [97]. The next evolutionary step involves the development of autonomous closed-loop systems. Here, ML algorithms integrated within the feedback control loop can optimize the device not only for a target wavelength but also for secondary performance metrics such as laser linewidth stability or output power. Reinforcement learning could further enable these systems to “learn” and adapt optimal control policies in response to changing operational conditions, paving the way for truly autonomous photonic subsystems [86]. Collectively, these advancements will foster the first generation of self-calibrating and self-optimizing intelligent photonic components, significantly enhancing system robustness and reducing operational overhead, thereby realizing the vision of “smart” photonics [98].

#### 6.2.3. Toward Fully Programmable Photonic Systems

The ultimate ambition for active FBG technology is its role as a foundational building block for fully software-defined photonic systems, offering unprecedented reconfigurability. This vision centers on the deployment of dense arrays of independently addressable active FBGs, which form the core of reconfigurable optical processors. Such programmable arrays can be dynamically configured to implement a wide range of functions—including finite impulse response (FIR) filters for microwave photonics [81,99], spectral shapers for advanced optical communications [100,101], and true time-delay lines for phased-array antennas [88,102]—all on a single, versatile hardware platform. This concept naturally extends to the broader architectural vision of a “Field-Programmable Photonic Gate Array (FPPGA)”. An FPPGA would comprise a generic optical chip containing gain sections, modulators, and programmable FBG-based filtering/interferometric units, which could be configured post-fabrication to emulate specific subsystems like a tunable laser, a microwave photonic filter, or a spectral processor [103,104,105]. Such programmable subsystems are also pivotal for next-generation reconfigurable optical computing modules. In neuromorphic computing, for example, tunable FBG arrays could implement reconfigurable weight banks within optical neural networks, enabling adaptive inference and on-chip learning capabilities [87,106].

#### 6.2.4. Quantum-Compatible Bragg Structures

The rapid advancement of quantum technologies from laboratory demonstrations toward practical systems creates a new and demanding frontier for FBG development, pushing performance specifications to extreme limits. A critical research direction involves engineering tuning mechanisms and materials for high-stability operation in cryogenic environments. Developing FBGs and their actuators to function with high precision and stability at temperatures as low as 4 K is essential for creating efficient interfaces with superconducting quantum processors. This necessitates a fundamental re-examination of material properties and may involve engineering specialized fibers or exploring cryogenically effective actuation principles [27,33,107]. Parallel to this, there is a growing need for Bragg structures specifically designed for quantum light manipulation. This entails the development of FBGs with extraordinary performance metrics tailored for quantum applications: ultra-narrow linewidths (sub-kHz) combined with ultra-high extinction ratios (exceeding 70 dB) for filtering pump noise from single-photon signals, and devices capable of precise wavelength matching for quantum frequency conversion in heterogeneous quantum networks [78,81,108]. Meeting the unique demands of quantum photonics will drive FBG technology toward unprecedented levels of precision and spectral purity.

### 6.3. Synthesis and Prospective Vision

The four pathways outlined above address different aspects of the performance matrix. Novel materials (graphene, PCMs) offer the most direct route to overcoming fundamental speed-power trade-offs, with potential for nanosecond-scale tuning at microwatt power levels [94,95]. AI-driven control provides a complementary approach to mitigate actuator imperfections without altering the physical tuning mechanism, particularly valuable for retrofitting existing systems [87,97]. Fully programmable photonic systems represent the long-term vision, requiring breakthroughs in integration density and fabrication scalability [88,103,104]. Quantum-compatible structures address the most demanding niche applications, where sub-kHz linewidths and cryogenic operation are non-negotiable [97,106]. For researchers entering the field, we recommend prioritizing materials-based innovations for fundamental performance leaps, while pursuing AI control for near-term system-level improvements. The convergence of these pathways—exemplified by AI-optimized metamaterial FBGs—represents the ultimate frontier for active wavelength control technology.

The future evolution of active FBG wavelength-control technology is inextricably linked to the broader megatrends of intelligentization, deep integration, and application-specific specialization. The interconnected pathways delineated—spanning novel material platforms, AI-empowered control, fully programmable systems, and quantum-optimized components—collectively herald a paradigm shift. This shift moves the role of FBGs from that of standalone, fixed-function components to that of integral, intelligent, and reconfigurable functional blocks within complex photonic systems. This trajectory, as evidenced by the vibrant and expanding research advancements discussed throughout this review, is paving the way for a new era of “software-defined photonics.” In this envisioned future, the propagation and properties of light can be manipulated with unparalleled agility, precision, and contextual awareness. Such capabilities promise to unlock transformative applications in areas like optical computing, distributed quantum networks, and adaptive sensing systems, which are currently in their nascent stages. Realizing this vision will demand sustained interdisciplinary collaboration, continually pushing the boundaries of what is possible with this remarkably versatile photonic element.

## 7. Conclusions

### 7.1. Summary of Key Findings

This review has provided a comprehensive and systematic examination of active wavelength control technology for fiber Bragg gratings (FBGs), charting its progression from fundamental tuning mechanisms to cutting-edge applications and future frontiers. The analysis establishes that the precise manipulation of the Bragg condition ($\lambda_{B}=2n_{eff}\Lambda$) through external fields has elevated FBGs from passive optical elements to versatile, programmable photonic core components.

The review systematically categorized and compared the fundamental tuning mechanisms—including mechanical, thermal, and optical methods—and detailed the key strategies developed to enhance their performance in terms of range, speed, and linearity. It then elucidated the transformative impact of these actively controlled FBGs across critical application domains: enabling wavelength-agile and narrow-linewidth fiber lasers, serving as reconfigurable filters and processors in microwave photonic systems, and emerging as indispensable tools in quantum information science and advanced biomedical imaging. Furthermore, the study critically analyzed the fundamental challenges constraining further advancement, such as performance trade-offs, control complexity, and integration bottlenecks. Finally, it outlined promising research directions focused on novel material platforms, AI-driven intelligent tuning, and quantum-optimized Bragg structures.

### 7.2. Main Contributions of This Review

The primary contribution of this review is the consolidation of active FBG wavelength control as a distinct and pivotal paradigm within modern photonics. By strictly delineating the scope to exclude passive sensing applications, this work provides the first dedicated and comprehensive framework for understanding FBGs as dynamic, programmable components. Specifically, it contributes by:

1. Unifying Diverse Technologies: Presenting a unified taxonomy and comparative analysis of tuning mechanisms and enhancement techniques, offering a clear reference for selecting appropriate strategies for specific application needs.
2. Connecting Applications with Technology: Demonstrating how specific technical advancements directly enable groundbreaking functionalities in disparate fields, from laser spectroscopy to quantum key distribution, thereby illustrating the technology’s cross-disciplinary value.
3. Providing a Forward-Looking Roadmap: Synthesizing current challenges into a coherent set of future research trajectories, offering actionable guidance for advancing the field towards intelligent, integrated, and application-specific photonic systems.

### 7.3. Limitations of the Study

While this review strives for breadth and depth, certain inherent limitations should be acknowledged. The literature search for this review was conducted in December 2025. Given the rapid evolution of the field, groundbreaking advances reported after the literature search cutoff date may not be included. Furthermore, constrained by the scope of a single article, the discussion of some highly specialized or nascent sub-fields, while highlighted, may not achieve the depth possible in a dedicated review. The focus on active control also necessarily meant that interesting work on the periphery of this core theme, or utilizing related but different technologies, may not have been covered.

### 7.4. Final Outlook

Looking ahead, active FBG wavelength control technology is advancing towards a new paradigm characterized by intelligentization, deep integration, and multi-functional convergence. Its evolution will be driven by the fusion of novel materials, artificial intelligence, and quantum photonics. Future research will focus on overcoming key challenges in tuning speed, energy efficiency, and stability under extreme conditions, with heterogeneous integration onto photonic integrated circuits being a critical milestone. As these advancements mature, actively controlled FBGs are poised to transition from programmable components to the core of “software-defined” photonic systems, playing an indispensable role as intelligent photonic primitives in next-generation optical communication, high-performance computing, and the quantum internet.

## Figures and Tables

**Table 1:** Summary and Comparison of Fundamental Tuning Mechanisms.

Tuning Mechanism	Primary Parameter Changed	Tuning Range	Response Speed	Precision/ Linearity	Key Advantages	Key Challenges
Axial Strain	Λ (Dominant)	Large (>40 nm)	Moderate (ms)	High/ Good	Large range, fast, linear	Mechanical reliability, hysteresis
Thermal	$n_{eff}$ (Dominant)	Moderate (3–5 nm)	Slow (s)	Very High/ Excellent	No moving parts, highly stable & precise	Slow, power- intensive
Optothermal	$n_{eff}$ (Via ΔT)	Moderate (5–10 nm)	Moderate (ms)	High/ Good	Non-contact, remote, precise	Speed limited by thermal diffusion
Transverse Load	$n_{eff}$ (Birefringence)	Small (<1 nm)	Moderate (ms)	Low/ Poor	Vector sensing capability	Spectral splitting, not pure tuning
Electro-Optic	$n_{eff}$	Very Small (<0.1 nm)	Fast (ns-ps)	Moderate	Ultra-high speed potential	Complex, costly, compatibility
Nonlinear Optical	$n_{eff}$ (Direct)	Very Small (<0.1 nm)	Fast (ns-fs)	Moderate	Ultra-fast, non-contact	High power required, complex
Hybrid	Λ & $n_{eff}$	Extensible	Depends on combination	Optimizable	Performance synergy, functionality	System complexity, control

Tuning range values are representative based on typical silica fiber FBGs at 1550 nm. Actual performance may vary with specific implementations.

**Table 2:** Overview of Key Techniques and Performance Enhancements.

Tuning Mechanism	Primary Techniques/Methods	Core Performance Enhancement
Mechanical Tuning	Lever structure amplification, elastic beam optimization (cantilever/simply supported beam), ultrasonic vibration	Tuning range expansion, response speed improvement, linearity enhancement
Thermal Tuning	Packaging with high-thermal expansion materials, optothermal effect (e.g., using MXene films)	Significant enhancement of thermal sensitivity, enables gradient temperature fields and non-uniform tuning
Interrogation & Control System	Tunable Fabry–Perot filter, FPGA-based digital signal processing	Improved system measurement accuracy and speed, enhanced real-time capability, temperature drift compensation
Special Structures & Fabrication Techniques	Femtosecond laser direct writing, composite beams and differential structures	Enables complex device structures, multi-parameter decoupling, and sensitivity improvement

## Data Availability

No new data were created or analyzed in this study. Data sharing is not applicable to this article.
